# RNA Transcripts in Human Ovarian Cells: Two-Time Cryopreservation Does Not Affect Developmental Potential

**DOI:** 10.3390/ijms24086880

**Published:** 2023-04-07

**Authors:** Yang Zhou, Wanxue Wang, Plamen Todorov, Cheng Pei, Evgenia Isachenko, Gohar Rahimi, Peter Mallmann, Frank Nawroth, Volodimir Isachenko

**Affiliations:** 1 Department of Obstetrics and Gynecology, Medical Faculty, Cologne University, 50931 Cologne, Germany; 2 Institute of Biology and Immunology of Reproduction of Bulgarian Academy of Sciences, 1113 Sofia, Bulgaria; 3 Center for Infertility, Prenatal Medicine, Endocrinology and Osteology, Amedes Medical Center MVZ Hamburg, 20095 Hamburg, Germany

**Keywords:** human, ovarian, cells, cryopreservation, mRNA sequencing, development, follicles, DEG, KEGG pathway and PPI analysis, gene ontology

## Abstract

Sometimes, for medical reasons, when a frozen tissue has already thawed, an operation by re-transplantation may be cancelled, and ovarian tissues should be re-frozen for transplantation next time. Research about the repeated cryopreservation of ovarian cells is rarely reported. It has been published that there is no difference in the follicle densities, proportions of proliferation of early preantral follicles, appearance of atretic follicles, or ultrastructural quality of frozen-thawed and re-frozen-rethawed tissue. However, the molecular mechanisms of a repeated cryopreservation effect on the developmental potential of ovarian cells are unknown. The aim of our experiments was to investigate the effect of re-freezing and re-thawing ovarian tissue on gene expression, gene function annotation, and protein–protein interactions. The morphological and biological activity of primordial, primary, and secondary follicles, aimed at using these follicles for the formation of artificial ovaries, was also detected. Second-generation mRNA sequencing technology with a high throughput and accuracy was adopted to determine the different transcriptome profiles in the cells of four groups: one-time cryopreserved (frozen and thawed) cells (Group 1), two-time cryopreserved (re-frozen and re-thawed after first cryopreservation) cells (Group 2), one-time cryopreserved (frozen and thawed) and in vitro cultured cells (Group 3), and two times cryopreserved (re-frozen and re-thawed after first cryopreservation) and in vitro cultured cells (Group 4). Some minor changes in the primordial, primary, and secondary follicles in terms of the morphology and biological activity were detected, and finally, the availability of these follicles for the formation of artificial ovaries was explored. It was established that during cryopreservation, the CEBPB/CYP19A1 pathway may be involved in regulating estrogen activity and CD44 is crucial for the development of ovarian cells. An analysis of gene expression in cryopreserved ovarian cells indicates that two-time (repeated) cryopreservation does not significantly affect the developmental potential of these cells. For medical reasons, when ovarian tissue is thawed but cannot be transplanted, it can be immediately re-frozen again.

## 1. Introduction

With the improvement in early diagnoses and treatments in oncology in the past three decades, survival rates of 5 years have increased significantly in both adults and children, reaching 65% and 83%, respectively [1]. Chemoradiotherapy has become one of the most critical cancer treatment approaches that make remission and significantly improve the prognosis. Still, they are usually highly toxic and harm the potential of fertility [2].

Many techniques for fertility preservation, such as cryopreservation of ovarian tissue, embryo, and oocyte, are proposed for female patients [3]. Among these techniques, ovarian tissue cryopreservation, which is safe and optimistic in terms of the recovery of the endocrine function and fertilization, is the first technique that is proposed to patients with malignant tumors who have the desire to preserve their fertility [4,5]. In addition, this technology is the only option for prepubertal female patients to preserve their fertility, because ovarian stimulation and oocyte collection are not available for them [6,7]. Ovarian tissue cryopreservation is also becoming an optimistic option for postponing menopause, especially in patients who are at high risk of cardiovascular disease, venous thromboembolism, dementia, and some kinds of hormone-dependent tumors and those who are not suitable for the hormonal replacement therapies [8,9,10]. Despite the great importance of the transplantation of cryopreserved ovarian tissue, the technique and skill should be further improved in order to increase its efficiency and safety [6,11,12].

The whole process of the cryopreservation of cells includes the following steps: (1) saturation of cells by permeable cryoprotectants, (2) cooling with crystallization (freezing), (3) thawing, and (4) the removal of cryoprotectants. Sometimes the term “freezing” is used in the sense of “cryopreservation”, which does not clearly reflect the essence of the process of solidification of solutions with biological objects at low temperatures, followed by thawing of those solutions and the removal of cryoprotectants. Conventional cryopreservation (slow freezing and quick thawing) is more efficient, which is accepted in the literature [13,14]. As conventional freezing and thawing techniques are the most widely used in the world, different kinds of research are being conducted to improve cryopreservation efficiency, focusing on the composition and concentration of cryoprotectant agents, freezing and thawing rates, and re-vascularization, [15,16].

A high proportion of viable follicles from different developmental stages is involved in research focusing on repeated cryopreservation [17]. Research about the repeated freezing and thawing of ovarian tissues, which may be applicable in some special clinical cases, is rarely reported.

Sometimes, for medical reasons, when frozen tissue is already thawed, an operation via re-transplantation may be cancelled, and if so, the ovarian tissues should be re-frozen for transplantation next time. Research about the repeated cryopreservation of ovarian cells is rarely reported. Some patients need to obtain the transplantation of ovarian cortical tissues that originate from the cryopreserved whole ovary due to unsuitable situations for whole ovary transplantation [18,19]. In this case, after the thawing of a whole ovary, only a part of this ovary will be used for the immediate re-transplantation of the patient. The rest of the ovary will be cryopreserved again, and in this case, avoiding double cryopreservation of ovarian cells is not possible.

Camille et al. reported that there is no difference in follicle densities, the proportions of proliferation of early preantral follicles, the appearance of atretic follicles, or the ultrastructural quality of one-time and two-time cryopreserved tissues [20]. However, the molecular mechanisms of repeated cryopreservation on the developmental potential of ovarian cells are unknown.

The aim of our experiments was to investigate the effects of re-freezing and re-thawing ovarian tissues on gene expression, gene function annotation, and protein–protein interaction. We also detected the morphological and biological activity of primordial, primary, and secondary follicles with the aim of using these follicles for the formation of artificial ovaries.

## 2. Results

### 2.1. Differential Expression Genes (DEGs)

**Figure 1:**
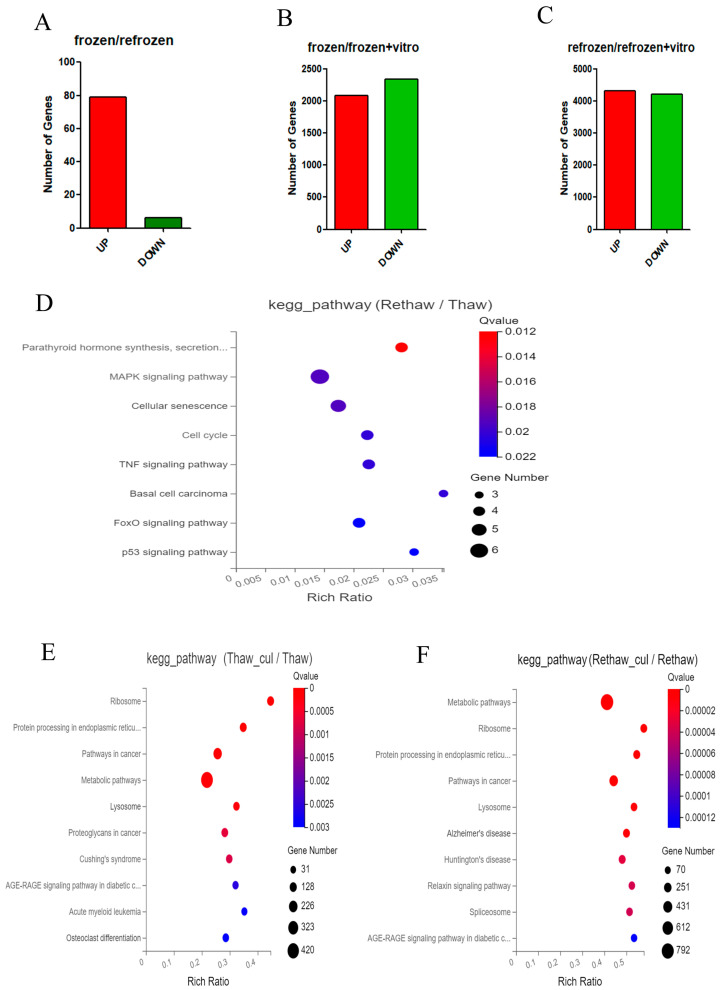
Visualization of Kyoto Encyclopedia of Genes and Genomes (KEGG) pathway enrichment for differential expression genes in cryopreserved ovarian cells in different comparisons. (**A**) Number of genes up- and downregulated in cells of Group 1 (one-time cryopreservation) and in cells of Group 2 (two-time cryopreservation). (**B**) Number of genes up- and downregulated in cells of Group 1 (one-time cryopreservation) and in cells of Group 3 (one-time cryopreservation and in vitro culture). (**C**) Number of genes up- and downregulated in cells of Group 2 (two-time cryopreservation) and in cells of Group 4 (two-time cryopreservation and in vitro culture). (**D**) Significant gene KEGG pathway in cells of Group 1 (one-time cryopreservation) and in cells of Group 2 (two-time cryopreservation). (**E**) Significant gene KEGG pathway in cells of Group 3 (one-time cryopreservation and in vitro culture). (**F**) Significant gene KEGG pathway in cells of Group 2 (two-time cryopreservation) and in cells of Group 4 (two-time cryopreservation and in vitro culture).

At the beginning of our research, a differential expression genes (DEGs) analysis was performed. A total of 85 genes were differentially expressed, and among them, 79 genes were upregulated, and 6 genes were downregulated in Group 2 (two-time cryopreservation) (re-frozen and re-thawed cells) and in Group 4 (two-time cryopreservation and in vitro culture) (re-frozen, re-thawed, and in vitro cultured cells) (Figure 1A). Then, the cells from Group 3 (one-time cryopreservation and in vitro culture) and Group 1 (one-time cryopreservation) were compared. It was established that there were 2084 genes that were upregulated and 2340 genes that were downregulated (Figure 1B). In Group 2 (two-time cryopreservation), 4307 genes were upregulated, and in Group 4 (two-time cryopreservation and in vitro culture), 4201 genes were downregulated (Figure 1C).

### 2.2. Kyoto Encyclopedia of Genes and Genomes (KEGG)

From the perspective of the KEGG pathway analysis, we gained deeper insight into DEGs.

DEGs in the cells in Group 1 (one-time cryopreservation) and in Group 2 (two-time cryopreservation) were mainly enriched in the pathways of parathyroid hormone synthesis, secretion, and action; MAPK signaling pathway; cellular senescence; cell cycle; TNF signaling pathway; basal cell carcinoma; FoxO signaling pathway; and p53 signaling pathway (Figure 1D).

DEGs in the cells of Group 1 (one-time cryopreservation) in comparison with DEGs in the cells of Group 3 (one-time cryopreservation and in vitro culture) were mainly enriched in the top five metabolic pathways: ribosome, protein processing in the endoplasmic reticulum, pathways in cancer, and lysosome (Figure 1E). Meanwhile, DEGs in the cells of Group 2 (two-time cryopreservation) in comparison with DEGs in the cells of Group 4 (two-time cryopreservation and in vitro culture) were mainly enriched in the same top five pathways (Figure 1F).

### 2.3. Gene Ontology

A gene ontology (GO) analysis was performed to evaluate the DEGs in the cells of four different groups. It analyzed the functions of the biological process (BP), the cellular component (CC), and the molecular function (MF).

**Figure 2:**
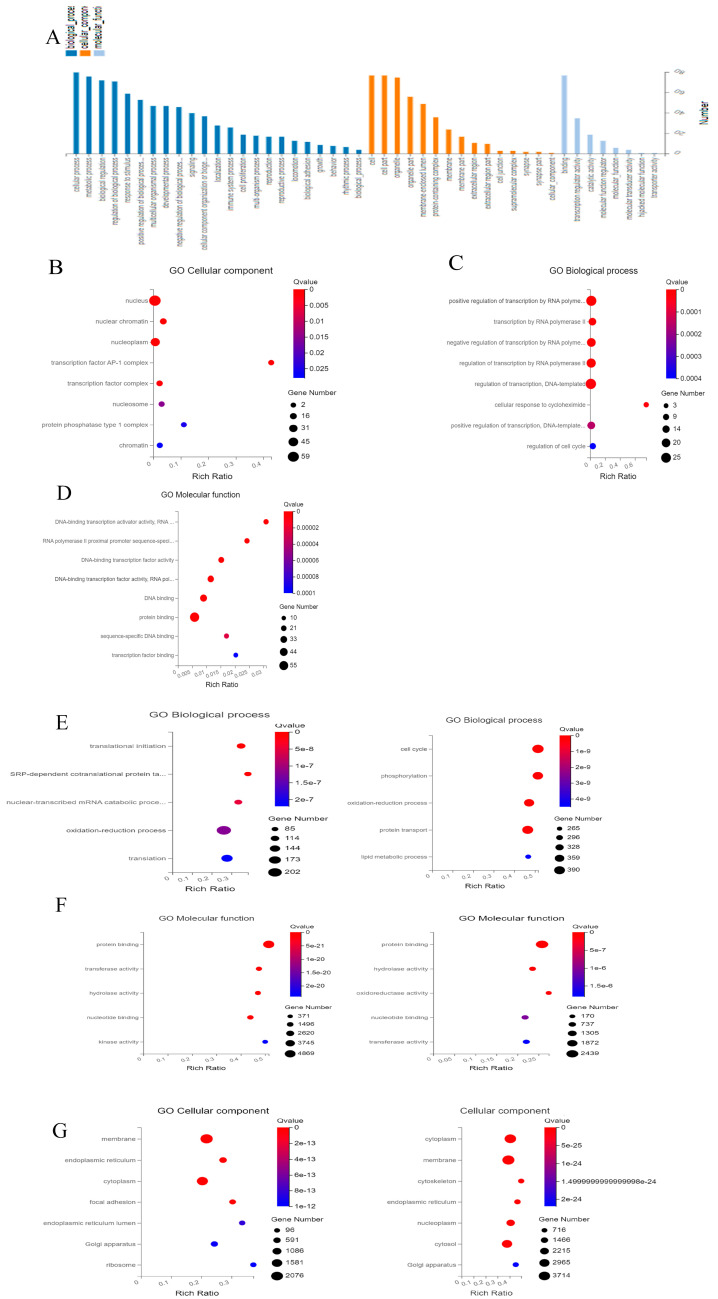
Visualization of gene ontology (GO) enrichment for differential expression genes in cryopreserved ovarian cells. (**A**) Gene ontology classification into functions of biological process (BP), cellular component (CC), and molecular function (MF) in cells of Group 1 (one-time cryopreservation) and Group 2 (two-time cryopreservation). (**B**–**D**) Top terms of cells from Group 1 (one-time cryopreservation) and Group 2 (two-time cryopreservation). (**E**–**G**) Top terms of cells from Group 3 (one-time cryopreservation and in vitro culture) and Group 4 (two-time cryopreservation and in vitro culture).

The DEGs in the cells of Group 1 (one-time cryopreservation) and Group 2 (two-time cryopreservation) were adopted for GO annotation classification (Figure 2A). It was established that a total of 46 GO terms were obviously annotated and 23 terms were included in the biological process, 15 were included in the cellular component, and 8 were included in the molecular function. They were then explicitly classified by DEGs into different functional categories or biological processes. A positive or negative regulation of transcription by an RNA polymerase II emerged as the most important and compelling enrichment term, which may primarily affect the potential function of ovarian cells. Secondly, the regulation of transcribed DNA, an enrichment term, influenced the response of ovarian tissue cells to cycloheximide (Figure 2B).

For the cellular component, differential transcripts were mainly associated with the nucleus, nuclear chromatin, and nucleoplasm (Figure 2C).

DNA-binding transcription activator activity and RNA polymerase II were the most significant identified enrichment terms in the molecular function categories. Secondly, the enrichment terms “RNA polymerase II proximal promoter sequence-specific DNA binding” and “DNA-binding transcription factor activity” were also statistically significant (Figure 2D).

A DEG analysis suggested that two-time cryopreservation had little effect on in vitro cultured-ovarian tissue. Data regarding the cells of Group 3 (one-time cryopreservation and in vitro culture) and cells of Group 4 (two-time cryopreservation and in vitro culture) were combined for an analysis (Figure 2E–G).

Translational initiation, SRP-dependent co-translational proteins targeting the membrane, nuclear-transcribed mRNA catabolic processes, nonsense-mediated decay, and the oxidation–reduction process were the top five terms in the cells of Group 3 (one-time cryopreservation and in vitro culture) (freezing and in vitro culture) in comparison with the parameters of the cells from Group 1 (one-time cryopreservation) (Figure 2E). At the same time, the cell cycle, phosphorylation, oxidation–reduction process, protein transport, and lipid metabolic process were the top five terms that were obviously annotated in cells of Group 4 (two-time cryopreservation and in vitro culture) (re-freezing and in vitro culture) in comparison with cells of Group 1 (one-time cryopreservation) (Figure 2E). Protein binding, hydrolase activity, nucleotide binding, transferase activity, and kinase activity were all annotated in the top five terms (Figure 2F). The viability of membranes, endoplasmic reticulum, and cytoplasm play main roles in cellular component categories (Figure 2G).

### 2.4. Protein–Protein Interactions (PPIs)

**Figure 3:**
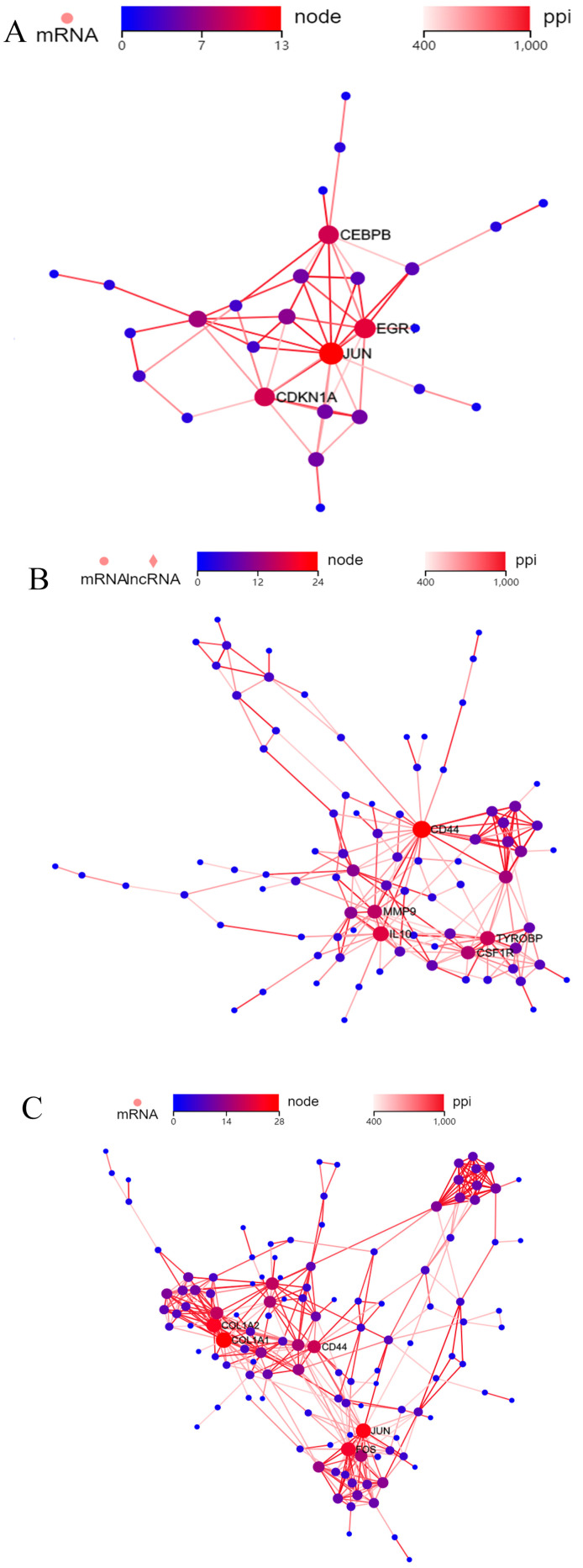
Expression of proteins in cryopreserved ovarian cells. (**A**) Top 5 proteins that were differentially expressed in cells of Group 1 (one-time cryopreservation) and Group 2 (two-time cryopreservation). (**B**) Top 5 proteins that were differentially expressed in cells of Group 3 (one-time cryopreservation and in vitro culture);.(**C**) Top 5 proteins that were differentially expressed in cells of Group 4 (two-time cryopreservation and in vitro culture).

Protein–protein interactions (PPIs) among DEGs were performed using the STRING database. The DEGs were first ranked for the determination of their *p*-value significance. Then, the top genes were adopted for a subsequent analysis. Then, the top nodes were finally identified, and the results showed that JUN, EGR1, CEBPB, and CDKN1A proteins became the central nodes in the cells of Group 1 (one-time cryopreservation) in comparison with the cells of Group 2 (two-time cryopreservation) (Figure 3A). In the cells of Group 3 (one-time cryopreservation and in vitro culture), CD44, IL10, MMP9, TYROBP, and CSF1R were the top five proteins that were differentially expressed (Figure 3B). In the cells of Group 4 (two-time cryopreservation and in vitro culture), FOS, JUN, COL1A1, COL1A2, and CD44 were the top five proteins with main roles (Figure 3C).

### 2.5. Intact and Active Follicles from Two-Time Cryopresrved Ovarian Tissues Can Be Used for the Formation of Artificial Ovaries

**Figure 4:**
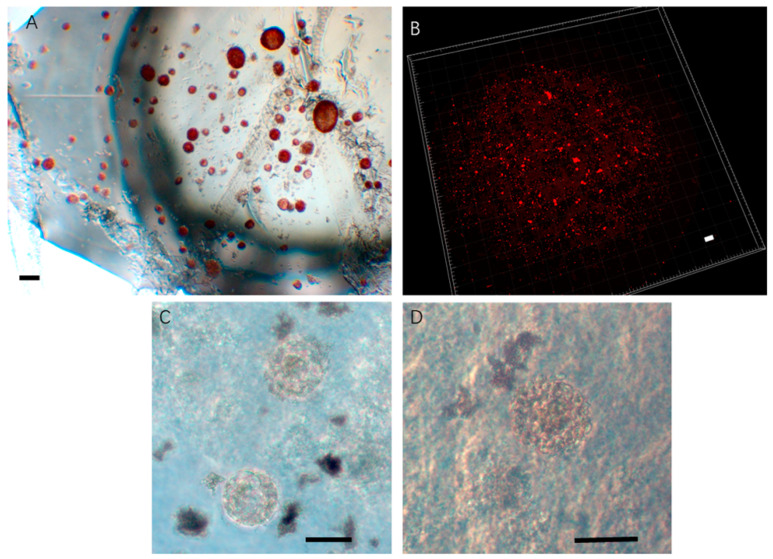
Follicles obtained from two-time cryopreserved ovarian tissues for construction of artificial ovaries. (**A**) Isolated live follicles stained by neutral red. (**B**) Artificial ovary stained by RedDot using Imaris 9.0. (**C**) Live primordial follicles from cryopreserved ovarian tissue in fibrin–thrombin complex. (**D**) Live primary follicles from cryopreserved tissue in fibrin–thrombin complex. Bar = 50 µm.

Nuclear and cytoplasmic staining vividly showed that the primordial and primary follicles were available after dissociation (Figure 4A). These follicles were collected into artificial ovarian scaffolds, which were prepared from fibrinogen and thrombin, and cultured in vitro for 7 days. The RedDot fluorescence marker was used for evaluation of living follicles (Figure 4B).

A quantitative analysis of the Imaris reconstruction indicated that the average loss of follicles was observed after 7 days of in vitro culture in Group 2 (two-time cryopreservation) in contrast with follicles of Group 1 (one-time cryopreservation). However, these differences were not statistically significant (13.42% decreasing, *p* = 0.13). Nuclear and cytoplasmic staining showed that those primordial follicles from Group 2 (two-time cryopreservation) were activated and developed into secondary follicles (Figure 4C).

## 3. Discussion

The basis for our experiments were the cases of surgical operation cancellations for ovarian tissue transplantation for clinical reasons. After thawing frozen ovarian tissue for transplantation, the question arises as to how the viability of this tissue will change after repeated cryopreservation. Periodically, our clinic records cases of cancellation of ovarian tissue transplantation when this tissue has already been thawed and the patient has undergone laparoscopy. Ovarian tissue cryopreservation is widely used in the preservation of female fertility. In our technology, we use the thawing of ovarian tissue in boiling water (100 °C). This element of technology is explained by the fact that in theoretical and applied cryobiology, there is a postulate that can be formulated as follows:

Any biological object that is cryopreserved using any of the existing cryopreservation methods should be thawed as quickly as possible. In our laboratory conditions, this is completed by thawing in boiling water. To increase the thawing speed, we use a magnetic stirrer. With the stirrer’s help, the thawing rate in boiling water increases by approximately 15%. In addition, immersing the cryo-tube with ovarian tissue in boiling water for 60 s sterilizes the surface of the tube, which is a plus under aseptic laboratory conditions.

For conventional freezing, there are two main factors that may cause cell and tissue damage. One is the ice crystal formation inside the cells and the other is excessive dehydration and shrinkage. These two damages are most likely to happen during cryopreservation. In this study, we adopted mRNA sequencing to analyze the transcription profiles of human ovarian tissues after repeated cryopreservation and to explore the effects of future in vivo cultures and the formation of artificial ovaries.

### 3.1. Differential Expressed Genes (DEGs)

Kirsten et al. reported that not only RNA quality and quantity but also RNA integrity and genetic expression profiles did not change much in the repeated (at least three times) cryopreservation of ovarian tumor tissues [21]. Our results demonstrated the feasibility of repeated ovarian tissue cryopreservation on gene expression and function levels. However, normal ovarian tissues with plenty of primordial and primary follicles are much more vulnerable to repeated freezing and thawing.

In our investigations, after the analysis of DEG, it was established that the two-time cryopreservation process rarely alters genes expression in ovarian cells. In comparison with the cells of Group 1 (one-time cryopreservation), in the cells of Group 2 (two-time cryopreservation), only 85 genes were differentially expressed among the whole mRNA genome, and most were identified as DEGs; 79 genes were highly upregulated, and 6 genes were downregulated. Among DEG, we concentrate on CYP11A1 (Cytochrome P450 Family 11 Subfamily A Member 1) and CYP19A1 (Cytochrome P450 Family 19 Subfamily A Member 1) genes that are coding estrogen synthetase, participating in the process of estrogen synthesis, and are both significantly downregulated in cells of Group 2 (two-time cryopreservation). Considering the important role of estrogen production for follicular development, it remains to be seen whether the low expression of these genes will affect the subsequent tissue function.

Recently, Bret et al. reported that cryopreserved human ovarian tissue is available for in vitro culture [22]. Previous studies have also verified that gonadotrophins, which are different kinds of growth factors, and nutrients, are affected in the in vitro maturation of oocytes [23]. Ovarian tissue culture for better follicle growth in vitro is an important method that can preserve the fertility of patients. At the same time, we compared the DEG in cells before and after in vitro culture. It was found that there were more alterations of differential genes, 4424 genes and 8508 genes, in the cells of Group 3 (one-time cryopreservation and in vitro culture) and Group 4 (two-time cryopreservation and in vitro culture), respectively.

In our study, it was firstly confirmed that both the one-time and two-time cryopreserved tissues were available for culturing with a gonadotrophin medium from the perspective of transcriptome profiles and functions. The cells of both these groups showed significant differences in gene expression after tissue culture. These differences were more prominent in Group 4 (two-time cryopreservation and in vitro culture), which indicates that the two-time cryopreservation does not affect the quality of the tissue’s activity. It was established that only three genes were differentially expressed, and they showed very few differences in genomic expression.

### 3.2. GO Terms Analysis

In our experiments, we adopted DEGs for a GO enrichment analysis and found that a series of basic cell activities (cellular process, metabolic process, biological regulation, and response to a stimulus) were all top enriched in GO terms of the biological process. The metabolism and regulation of related genes in ovarian cells were more susceptible in Group 2 (two-time cryopreservation), but related genes involved in these biological processes did not fundamentally affect follicle development, and they had little effect on fertility.

Protein binding, hydrolase activity, nucleotide binding, transferase activity, and kinase activity were similar in the cells of Group 1 (one-time cryopreservation) and Group 2 (two-time cryopreservation).

### 3.3. What Is KEGG Pathways Analysis?

Parathyroid hormone synthesis, secretion, and action, MAPK signaling pathways, cellular senescence, cell cycles, TNF signaling pathways, basal cell carcinoma, FoxO signaling pathways, and p53 signaling pathways were changed after double cryopreservation. The MAPK pathway played central roles in coordinating the follicle and oocyte development and stress response, which were then changed in cryopreserved (vitrified-warmed) mouse ovaries in comparison with the parameters of fresh ovaries [24,25].

P53 is a senescence marker that was downregulated after cryopreservation in human ovarian tissues [26]. Our research indicates that the process of cryopreservation (freezing and thawing) would accelerate the senescence of ovarian tissues.

We categorized significantly correlated pathways in Group 3 (one-time cryopreservation and in vitro culture) and Group 4 (two-time cryopreservation and in vitro culture) in comparison with the cells of Group 1 (one-time cryopreservation) and Group 2 (two-time cryopreservation). Most of these genes fell into metabolic pathways: ribosomes, protein processing in the endoplasmic reticulum, pathways in cancer, and lysosomes in both groups. Our results show that tissues in an in vitro culture involve a series of basic metabolic pathways, and this process was not affected by the two-time cryopreservation.

### 3.4. PPI Analysis

In our research, a DEG was adopted in the cells of Group 1 (one-time cryopreservation) and Group 2 (two-time cryopreservation) to perform protein–protein interactions.

EGR1 is a nuclear protein-encoded gene, and it usually functions as a transcriptional regulator. The aberrant expression of EGR1 is associated with the disease of ischemia [27], and previous research showed that it was upregulated in the surroundings of hypoxia [28].

In our research, EGR1 was also significantly upregulated in the tissues of Group 2 (two-time cryopreservation), and our results suggest that hypoxia occurred in the process of two-time cryopreservation. It is beneficial to consider increasing the oxygen content in the environment of the freeze-storage solution to optimize the protocol.

CEBPB could downregulate CYP19A1 expression and decrease 17-beta estradiol levels in the buffalo granulosa cell [29]. When combined with the previous findings, we speculated that the CEBPB/CYP19A1 pathway may be involved in the regulation of estrogen during cryopreservation.

CD44 was the main PPI connection, and it was significantly upregulated in the cells from Group 1 (one-time cryopreservation), Group 2 (two-time cryopreservation), Group 3 (one-time cryopreservation and in vitro culture), and Group 4 (two-time cryopreservation and in vitro culture). CD44 is a receptor on the surface of cells that functions in cell–cell interactions, cell apoptosis, adhesion, and migration, helping them to sense and respond to tissue microenvironment changes [30,31]. In addition, CD44 is an important stem cell marker and a critical regulator of cancer cells [32], and it also participates in the regulation of myoblast behaviors during the process of embryogenesis [33]. In our research, it was established that CD44 plays a crucial role in the regulation of cryopreserved ovarian tissue.

## 4. Materials and Methods

### 4.1. Design of Experiments

A total of twelve samples were distributed into four groups: one-time cryopreserved (frozen and thawed) cells (Group 1), two-time cryopreserved (re-frozen and re-thawed after first cryopreservation) cells (Group 2), cells one-time cryopreserved (frozen and thawed), and in vitro cultured after thawing (Group 3), and cells two-time cryopreserved (re-frozen and re-thawed after first cryopreservation) and in vitro cultured after second cryopreservation (Group 4 )(two-time cryopreservation and in vitro culture).

### 4.2. Cell Collection and Cryopreservation (Freezing and Thawing)

Except where otherwise stated, all chemicals were obtained from Sigma (Sigma Chemical Co., St. Louis, MO, USA). The study was performed in accordance with the Declaration of Helsinki. Ethical permission was approved by the Ethics Committee at the University Hospital of Cologne and by the Bulgarian Ethics Committee. Informed consent was obtained from all subjects involved in the study.

Cryopreservation of ovarian tissue (pieces) was performed according to our previously published protocol (14, 15, and 34). On the day of freezing, pieces of ovarian tissue were placed at room temperature in 20 mL freezing medium composed of basal medium supplemented with 6% dimethyl sulfoxide, 6% ethylene glycol, and 0.15 M sucrose. Then, pieces were put into standard 5 mL cryo-vials (Thermo Fisher Scientific, Rochester, NY, USA), which were previously filled by freezing medium and frozen in IceCube 14S freezer (SyLab, Neupurkersdorf, Austria). The cryopreservation program was as follows: (1) the starting temperature was −6 °C; (2) samples were cooled from −6 to −34 °C at a rate of 0.3 °C/min; and (3) at −34 °C cryo-vials were plunged into liquid nitrogen. The freezing protocol for cryopreservation of this ovarian tissue included an auto-seeding step at −6 °C.

Thawing of tissue was achieved by holding the vial for 30 s at room temperature, followed by immersion in a 100 °C (boiling) water bath for 60 s, and expelling the contents of the vial into the solution for the removal of cryoprotectants. The exposure time in the boiling water was visually controlled by the presence of ice in the medium; as soon as the ice reached 2 to 1 mm apex, the vial was removed from the boiling water, at which point the final temperature of the medium was between 4 and 10 °C. Within 5 to 10 s after thawing, the pieces from the cryo-vials were expelled into 10 mL thawing solution (basal medium containing 0.5 M sucrose) in a 100 mL specimen container (Sarstedt, Nuembrecht, Germany).

### 4.3. Confocal Microscopy

Confocal microscopy investigations were performed using LSM 710 microscope (Carl Zeiss, Jena, Germany) with a 3D-imaging setup comprising tile scanning and a transmission depth from 200 to 300 µm. Photographs were taken at 2 µm intervals. The original microscopy data were analyzed using Imaris 9.0 software. Imaris imaging reconstruction and analysis were performed using the “Surpass” and “3D view” modes. The threshold was changed to improve the accuracy of follicle identification. RedDot fluorescence staining was used to detect the live cells.

### 4.4. Artificial Ovary and Imaging

The construction of artificial ovary was performed according to our previously published protocol, with fibrin encapsulation and follicle isolation using Tumor Dissociate Enzyme (TDE) [34].

TISSEEL Fibrin Sealant (Baxter International Inc., Deerfield, IL, USA) was used to encapsulate the isolated follicles. Fibrinogen and thrombin at final concentrations of 45.5 mg/mL and 10 IU/mL, respectively, are desirable for optimal encapsulation. Both components were quickly mixed in an Eppendorf tube using a pipette and were then vortexed. The follicle suspension (30 to 50 follicles for one artificial ovary) was then added dropwise (30 µL) to form a nearly gelatinous mixture. The mixture was then dropped into a Petri dish designed for live cell imaging (WillCo Wells B.V, Amsterdam, The Netherlands). Follicles were cultivated in alpha-modified Eagle’s minimum essential medium (α-MEM, Gibco BRL; Life Technologies, Carlsbad, CA, USA) supplemented with 15% fetal calf serum, 2 mmol L-glutamine (Gibco BRL), insulin–transferrin–sodium selenite supplement (ITSE; Sigma-Aldrich, St. Louis, MO, USA), ascorbic acid (50 µg/mL), 100 IU/mL penicillin, 0.1 mg/mL streptomycin, and 300 mIU/mL human recombinant follicle stimulation hormone (Gonal F^®^; Serono Pharma GmbH, Munich, Germany) in a humidified incubator in a 5% CO_2_ atmosphere at 37 °C. Half the volume of the medium was changed every 48 h and all the artificial ovary samples were cultured for 7 days.

### 4.5. Sequencing and Data Extraction

Twelve ovarian tissue samples were used for RNA extraction. We detected which cells were used for library preparation with an Illumina compatible kit and sequencing by the DNBSEQ platform [19]. The raw data of this project (in fq.gz format) can be found in the Sequence Read Archive of the National Center for Biotechnology Information (Project No: PRJNA932020 (http://www.ncbi.nlm.nih.gov/bioproject/932020, accessed on 7 February 2023). Raw data are the original data of transcriptome sequencing.

The original sequenced reads, or raw reads, obtained by sequencing contain low-quality reads with adapters. In order to ensure the quality of information analysis, raw reads must be filtered to obtain clean reads. Subsequent analysis is based on clean reads. The filtering procedure was as follows: (1) removing reads containing adapters; (2) removing reads containing N > 10% (N represents bases that could not be determined); and (3) removing low-quality reads. The Q score (quality value) of >50% of the bases of the read is ≤5.

In order to avoid low-quality reads and reads with adapters from raw data, raw reads were filtered, and clean reads were obtained. The filtering procedure was as follows: (1) removing reads containing adapters; (2) removing reads containing N > 10% (N represents bases that could not be determined); and (3) removing low-quality reads. The Q score (quality value) of >50% of the bases of the read is ≤5.

### 4.6. Differential Gene Expression

Gene expression was investigated by the abundance of transcripts (sequencing counts) that mapped to genomes or exons. Read matter is proportional to genetic expression level, genetic size, and sequencing depth. Anticipating the variety of pieces per kilobase of transcript series per million base pairs sequenced (FPKM) is the most typical method of estimating gene expression levels, which takes into consideration the impact of both sequencing depth and gene size on fragment counts.

Raw data (raw reads) in FASTQ format were first run via internal Perl scripts. Overall, the downstream evaluations were based on clean, top-quality data. The GSEA tool (http://www.broadinstitute.org/gsea/index.jsp, accessed on 30 June 2022).

Gene ontology (GO), Kyoto Encyclopedia of Genes and Genomes (KEGG), Reactome, Disease Ontology (DO), and DisGeNET were used for epigenetic annotation of the differential genes. GO analysis has been widely used in the field of bioinformatics, which covers three aspects of biology: cellular components, molecular functions, and biological processes. KEGG is an annotation for understanding advanced functions and utilities of cells and organisms. Reactome analysis constructed biological molecular signaling pathway annotation in this study.

Differential genes from each group were also analyzed using protein–protein interaction (PPI), which could systematically analyze the interaction relationships of a large number of proteins in biological systems, which is important for understanding how proteins work in samples, the response mechanisms of biological signals, and energy-matter metabolism in specific freezing conditions, as well as the functional connections between proteins.

## 5. Conclusions

This analysis of the gene expression in cryopreserved ovarian cells indicates that two-time (repeated) cryopreservation does not significantly affect the developmental potential of these cells. For medical reasons, when ovarian tissue is thawed but cannot be transplanted, it can be immediately re-frozen.

## Data Availability

The raw data of RNA-seq can be downloaded at “Sequence read archive” on National Center for Biotechnology Information (http://www.ncbi.nlm.nih.gov/bioproject).
